# *bioA* mutant of *Mycobacterium tuberculosis* shows severe growth defect and imparts protection against tuberculosis in guinea pigs

**DOI:** 10.1371/journal.pone.0179513

**Published:** 2017-06-28

**Authors:** Ritika Kar, Prachi Nangpal, Shubhita Mathur, Swati Singh, Anil K. Tyagi

**Affiliations:** 1 Department of Biochemistry, University of Delhi South Campus, Benito Juarez Road, New Delhi, India; 2 Vice Chancellor, Guru Gobind Singh Indraprastha University, Dwarka, New Delhi, India; Public Health England, UNITED KINGDOM

## Abstract

Owing to the devastation caused by tuberculosis along with the unsatisfactory performance of the Bacillus Calmette–Guérin (BCG) vaccine, a more efficient vaccine than BCG is required for the global control of tuberculosis. A number of studies have demonstrated an essential role of biotin biosynthesis in the growth and survival of several microorganisms, including mycobacteria, through deletion of the genes involved in *de novo* biotin biosynthesis. In this study, we demonstrate that a *bioA* mutant of *Mycobacterium tuberculosis* (MtbΔ*bioA*) is highly attenuated in the guinea pig model of tuberculosis when administered aerogenically as well as intradermally. Immunization with MtbΔ*bioA* conferred significant protection in guinea pigs against an aerosol challenge with virulent *M*. *tuberculosis*, when compared with the unvaccinated animals. Booster immunization with MtbΔ*bioA* offered no advantage over a single immunization. These experiments demonstrate the vaccinogenic potential of the attenuated *M*. *tuberculosis bioA* mutant against tuberculosis.

## Introduction

The vaccine Bacillus Calmette–Guérin (BCG), an attenuated strain of *Mycobacterium bovis*, has been widely administered since 1921. BCG protects children against severe forms of tuberculosis (TB) but demonstrates highly variable efficacy (0–80%) against adult pulmonary tuberculosis in different field trials with poor efficacy in high TB burden countries, in general [1–5]. There is a clear need for a new effective TB vaccine and a number of candidate vaccines based on several different approaches are currently being evaluated in clinical trials [6]. However, one of the most advanced TB vaccine candidate that attempted to boost the efficacy of BCG was unable to induce any additional efficacy over BCG in a recent human clinical trial [7]. One promising approach of vaccine development against TB is the generation of attenuated *Mycobacterium tuberculosis* mutant strains. Vaccine strains based on attenuated *M*. *tuberculosis* mutants harness the benefits of close antigenic similarity with the disease-causing organism. A study comparing BCG and *M*. *tuberculosis* substrains has shown that a large number of T-cell epitopes are missing from BCG substrains but are present in all five *M*. *tuberculosis* strains employed for the comparative analysis [8]. Technological advancements have accelerated site-specific genetic modifications in mycobacteria. In addition, identification of virulence factors as well as essential biosynthetic pathways of *M*. *tuberculosis* has enabled the generation of rationally attenuated *M*. *tuberculosis* mutant strains. Several *M*. *tuberculosis* mutant strains have been shown to be safe and attenuated while immunization with these strains have resulted in the generation of varying degree of protection against TB in animal models when compared with the protection afforded by BCG [9–16]. Though several attenuated *M*. *tuberculosis* mutant based vaccine strains have been generated, MTBVAC (*M*. *tuberculosis* mutant with deletions in the genes *phoP* and *fadD26*) is the first and the only live attenuated *M*. *tuberculosis* vaccine strain that is being assessed in human clinical trials [17]. Thus, further efforts are needed towards the development of novel attenuated *M*. *tuberculosis* strains and evaluation of their efficacy against tuberculosis.

Biotin is central to cell metabolism in both prokaryotes and eukaryotes. It is an essential cofactor for various enzymes catalyzing the transfer of CO_2_ during carboxylation, decarboxylation and transcarboxylation reactions. Bacteria and plants can synthesize biotin *de novo* but mammals do not contain the enzymes required for biotin synthesis and depend on its supplementation through diet or gut microbiota [18–20]. BioA (7,8-diaminopelargonic acid synthase) catalyzes the conversion of KAPA (7-keto-8-aminopelargonic acid) to DAPA (7,8-diaminopelargonic acid) in the biotin biosynthesis pathway [21]. The precursor for biotin biosynthesis is a pimeloyl moiety that may be obtained from different sources but the final four enzymatic steps are conserved between all biotin-synthesizing organisms [19, 22]. Since biotin biosynthetic pathway is unique to bacteria and plants, it has been an important target for developing inhibitors against *M*. *tuberculosis* [23–25]. Several evidences support the essentiality of biotin biosynthesis for the *in vitro* as well as *in vivo* survival of mycobacteria. Firstly, the *bioA* gene mutant of *M*. *smegmatis* was found to be auxotrophic in nature and depended on the *de novo* biosynthesis of biotin for optimal growth and survival in the stationary phase even in the presence of biotin in the media [26]. Secondly, a hallmark study employing transposon mutagenesis identified genes of biotin biosynthetic pathway (*bioF*, *bioA* and *bioB*) to be essential for the survival of *M*. *tuberculosis* in mice which suggested that *M*. *tuberculosis* does not have access to exogenous biotin *in vivo* [27]. Thirdly, an *M*. *marinum* biotin auxotroph was shown to be growth defective *in vitro* in macrophages as well as severely attenuated *in vivo* in a zebrafish model of tuberculosis [28]. Importantly, transporters that facilitate the uptake of exogenous biotin have not been identified in mycobacteria but biotin, when supplied in copious amounts in the growth media, seem to enter the bacilli by passive diffusion or by yet unidentified mechanism [19, 29]. The importance of BioA was substantiated in a recent study by Park *et al*., wherein *bioA* gene mutant of *M*. *tuberculosis* was constructed and this *M*. *tuberculosisΔbioA* strain was found to be auxotrophic for biotin [30]. Further, Park *et al*. evaluated the biotin biosynthetic pathway as a drug target against *M*. *tuberculosis* and demonstrated that BioA was essential for the establishment of infection as well as for the persistence of *M*. *tuberculosis* in mice [30]. However, certain features of tuberculosis in mice are quite distinct from humans and may have significant implications on vaccine evaluation and pathological studies. Most commonly used mice strains, C57BL/6 and BALB/c are inherently resistant to *M*. *tuberculosis* infection and do not develop severe pathology as seen in human TB [31]. Certain CD1 isoforms known to play a noteworthy role in immune response against TB are not found in mice but are present in guinea pigs [32, 33]. Also, granulomas in mice are not hypoxic and do not caseate unlike in human TB [34, 35]. Moreover, there are studies where vaccines exhibiting promise in the murine model failed when tested in guinea pigs [36–38]. Manifestation of TB in guinea pigs and humans bear a large number of similarities [39]. Guinea pigs are vulnerable to infection with a few inhaled *M*. *tuberculosis* bacilli and demonstrate many aspects of human TB such as caseating and hypoxic granulomas, pulmonary lesions of both primary and hematogenous origin, presence of Langhans multinucleated giant cells, robust delayed-type hyper-sensitivity (DTH) response and dissemination of infection [40–43]. Guinea pigs do not develop latency, rather they develop chronic progressive disease and lung tissue damage leading to weight loss and death, making this model ideal for studying TB associated pathology [40, 44, 45]. Guinea pig model is the gold standard for TB vaccine evaluation. Owing to the susceptibility to *M*. *tuberculosis* infection and consistent efficacy demonstrated following BCG vaccination, guinea pig model is accepted as a more stringent model for efficacy testing than mice.

In this study, we constructed a *bioA* (Rv1568) mutant of *M*. *tuberculosis* (MtbΔ*bioA*) and demonstrated its attenuation in the highly susceptible guinea pig model of tuberculosis following administration via the aerosol as well as intradermal route. Subsequently, the MtbΔ*bioA* strain was evaluated in single and booster immunization protocol for its ability to protect against virulent *M*. *tuberculosis* challenge in the guinea pig model of experimental tuberculosis.

## Materials and methods

### Bacterial strains and culture conditions

Bacterial strains and plasmids employed in this study are listed in Table 1. Mycobacterial strains were grown in Middlebrook (MB) 7H9 media (BD Difco) supplemented with 0.5% glycerol, 1X ADC (albumin-dextrose-catalase, BD Difco) and 0.05% Tween 80 or on MB7H11 agar (BD Difco) supplemented with 1X OADC (oleic acid-albumin-dextrose-catalase, BD Difco) and 0.5% glycerol. Cultures were grown at 37°C with constant shaking at 200 rpm. Agar plates were incubated at 37°C for 3–4 weeks. For infection and vaccination purposes, mycobacterial cultures in mid-log phase were resuspended in phosphate-buffered saline (PBS) and frozen in aliquots, until further use. The colony-forming units of the PBS stocks were determined by plating appropriate dilutions in duplicates on supplemented MB7H11 agar. For modified 7H9, 0.5 g ammonium sulfate, 0.5 g L-glutamic acid, 0.1 g sodium citrate, 1 mg pyridoxine, 2.5 g disodium phosphate, 1.0 g monopotassium phosphate, 0.04 g ferric ammonium citrate, 0.05 g magnesium sulfate, 0.5 mg calcium chloride, 1 mg zinc sulfate, 1 mg copper sulfate were added per litre of media and supplemented with 0.2% glycerol, 1X ADC (BD Difco) and 0.05% Tween 80.

**Table 1:** Bacterial strains, plasmids and primers employed in this study.

**Strains**	**Description**	**Reference**
*E*.*coli* XL-1 Blue	*endA*1 *gyrA*96 (*nalR*) *thi*-1 *recA*1 *relA*1 *lac glnV*44 F' [::Tn10 *proAB* + *lacIq* Δ (*lacZ*) M15] *hsdR*17 (rK- mK+)	Stratagene, Heidelberg, Germany
*E*.*coli* HB101	F-(*gpt-proA*) 62 *leuB*6 *glnV*44 *ara*-14 *galK*2 *lacY*1 (*mcrC-mrr*) *rpsL*20 (Strr) *xyl*-5 *mtl*-1 *recA*13	Life Technologies, CA, USA
*M*. *tuberculosis* H37Rv	Virulent strain of *M*. *tuberculosis*	Dr. J. S. Tyagi, AIIMS, New Delhi, India
*M*. *tuberculosis* H37Rv/pJV53	*M*. *tuberculosis* H37Rv recombineering strain harbouring pJV53 and expressing recombineering proteins gp60 and gp61	[46]
MtbΔ*bioA*	*M*. *tuberculosis* H37Rv *bioA* mutant	This study
MtbΔ*bioA*.comp	*M*. *tuberculosis* H37Rv *bioA* mutant complemented with full length *bioA* gene in extrachromosomal plasmid	This study
*M*. *tuberculosis* Erdman	Virulent strain of *M*. *tuberculosis*	Dr. J. S. Tyagi, AIIMS, New Delhi, India
BCG Danish	Vaccine strain against tuberculosis	BCG laboratories, Chennai, India
**Plasmids**	**Description**	**Reference**
pJV53	Mycobacterial- *E*. *coli* shuttle vector encoding recombineering proteins gp60 and gp61	[47]
pYUB854	Mycobacterial cosmid vector with hygromycin resistance gene cassette flanked by resolvase sites and multiple cloning sites	[48]
pYUBΔ*bioA*	pYUB854 with hygromycin resistance cassette flanked with *bioA* amplicons I and II	This study
pSD5.hsp65	Mycobacterial- *E*. *coli* shuttle expression vector containing hsp65 promoter	[49, 50]
pVR1	A pSD5 derivative containing the chloramphenicol resistance gene under mycobacterial *trrn* promoter	[51]
pVR1.*bioA*	pVR1 carrying the full length mycobacterial *bioA* gene under hsp65 promoter	This study
**Primers**	**Sequence**	**Reference**
BioA-AI-F	5' -gatatcactagtatgccaatagcgttcgctga- 3'	This study
BioA-AI-R	5' -agtactagatcttttaaagcggtccaccaggagctcat- 3'	This study
BioA-AII-F	5' -agtactggtacctttaaatccatcggcgtcatcgaa- 3'	This study
BioA-AII-R	5' -gatatccttaaggcgtcaccacaaccc- 3'	This study
F-*bioA*	5' -gattatcatatgggatccatggctgcggcgactggc- 3'	This study
R-*bioA*	5' -cttatatcctcgagtcatggcagtgagcctac- 3'	This study

*Escherichia coli* strains HB101 (Life Technologies) and XL-1 Blue (Stratagene) were used to clone specific DNA segments into pYUB854 and pVR1 plasmids, respectively (Table 1). *E*. *coli* cells were grown in Luria Bertani (LB) broth at 37°C with constant shaking at 200 rpm. When required, kanamycin (25 μg/ml), chloramphenicol (30 μg/ml) and hygromycin (50 μg/ml for mycobacteria and 150 μg/ml for *E*. *coli*) were added to the growth media.

### Disruption of the *bioA* gene in *M*. *tuberculosis*

For the generation of MtbΔ*bioA* mutant, internal 916 bp segment of the *bioA* gene was deleted in the genome of *M*. *tuberculosis* H37Rv and was replaced with the hygromycin resistance cassette by employing a linear allelic exchange substrate (AES). To generate the linear Δ*bioA*::*hyg* AES, two set of primers were designed to amplify amplicon I and amplicon II (Table 1). The 729 bp amplicon I consisted of 200 bp of 5′ proximal end of the *bioA* gene and 529 bp of immediate upstream region of the *bioA* gene. The 648 bp amplicon II comprised of 198 bp of 3′ distal end of the *bioA* gene and 450 bp of the region immediately downstream of the *bioA* gene. BioA is encoded by the first gene of an operon consisting of three downstream genes (*bioF1*, *bioD* and Rv1571). Hence, the amplicons were designed such that homologous recombination would result in deletion of internal 916 bp segment of the *bioA* gene leaving behind ~200 bp region at the 5′ and 3′ end of the *bioA* gene along with its upstream and downstream regions intact. Moreover, the hygromycin resistance cassette was inserted in reverse orientation to the *bioA* gene. The amplicon I and amplicon II were PCR amplified and cloned into the vector pYUB854 at *Bgl*II/*Spe*I and *Kpn*I/*Afl*II restriction sites, respectively, flanking the hygromycin resistance cassette to generate pYUBΔ*bioA* (Table 1). Subsequently, the 3.4 kb linear AES (*ΔbioA*::*hyg AES*) was excised out of the vector pYUBΔ*bioA* by *Afl*II/*Spe*I restriction digestion and electroporated into *M*. *tuberculosis* H37Rv/pJV53 as described earlier [47] to generate the *bioA* mutant of *M*. *tuberculosis*.

### Construction of complemented strain of MtbΔ*bioA*

The pJV53 recombineering plasmid employed for the generation of the mutant was removed from the MtbΔ*bioA* strain prior to the complementation. For this, MtbΔ*bioA* was cultured in MB7H9 media in the absence of kanamycin (selection marker for pJV53) and appropriate dilutions of tertiary culture were plated on MB7H11 agar containing hygromycin. Individual colonies obtained after the incubation of agar plates for 3–4 weeks at 37°C were screened for the loss of pJV53 by patching on MB7H11 agar plates with or without kanamycin (S1 Fig). In this study, 80% of the colonies screened showed kanamycin sensitive phenotype and thus demonstrated the loss of pJV53 from MtbΔ*bioA*.

For complementation of MtbΔ*bioA* strain, 1.3 kb full-length *bioA* gene from mycobacteria was cloned downstream to hsp65 promoter in the vector pSD5.hsp65 resulting in the generation of pSD5.hsp65-*bioA* (Table 1). The hsp65-*bioA* segment was further subcloned into the *Xba*I and *Mlu*I sites of the mycobacterial shuttle vector pVR1, to generate pVR1.*bioA* (Table 1). The plasmid pVR1.*bioA* was subsequently electroporated into MtbΔ*bioA* strain to generate the complemented strain, MtbΔ*bioA*.comp. The transformants were selected on MB7H11 agar plates supplemented with 30 μg/ml chloramphenicol followed by incubation at 37°C for 3–4 weeks. For immunoblot analysis, 10 μg cell lysate of *M*. *tuberculosis* H37Rv, MtbΔ*bioA* mutant and MtbΔ*bioA*.comp strains were subjected to SDS-PAGE (sodium dodecyl sulfate–polyacrylamide gel electrophoresis) in 12.5% polyacrylamide gel. Immunoblotting was performed using anti-BioA rabbit polyclonal antiserum (kindly provided by Dr. Dirk Schnappinger and Dr. Sabine Ehrt, Weill Cornell Medical College, New York) [30] at a dilution of 1:2000 followed by addition of goat anti-rabbit secondary antibody conjugated to horseradish peroxidise (Jackson Immuno Research Inc.). Blot was detected with 1 mg/ml of 3’,3’–diaminobenzidine in phosphate buffered saline containing 0.3% H_2_O_2_.

### Experimental animals

Female guinea pigs (Dunkin-Hartley) of 200–300 g were procured from Disease Free Small Animal House Facility, Lala Lajpat Rai University, Hisar, India. The animals were housed in individually ventilated cages under standardized conditions in a Biosafety level III facility at University of Delhi South Campus (UDSC), New Delhi, India and were provided with food and water *ad libitum*. Animals were allowed to acclimate, following which they were randomized to different experimental groups.

### Ethics statement

All guinea pig experiments included in this study were reviewed and approved by the Institutional Animal Ethics Committee of University of Delhi South Campus, New Delhi, India (Ref. No. 9/IAEC/AKT/Biochem/UDSC/29.08.2014 and Ref No. 26/IAEC/AKT/Biochem/UDSC/17.8.2015). All animals were routinely cared for according to the guidelines of CPCSEA (Committee for the Purpose of Control and Supervision of Experiments on Animals), India and all efforts were made to ameliorate animal suffering. Guinea pigs were intradermally vaccinated by injecting 100 μl of suspension and were euthanized by CO_2_ asphyxiation, whenever required, during daytime in the Biosafety level III facility. No animals died prior to the experimental timepoints.

### Evaluation of attenuation and *in vivo* growth kinetics of MtbΔ*bioA* in guinea pigs

To evaluate the extent of attenuation of the MtbΔ*bioA* strain, guinea pigs in groups of six were infected via aerosols with ~10–30 bacilli of *M*. *tuberculosis* H37Rv or MtbΔ*bioA* or MtbΔ*bioA*.comp in an aerosol chamber (A4224—full body inhalation exposure system, Glas-Col Inc., USA). Animals were euthanized by CO_2_ asphyxiation at 3, 6 and 12 weeks post infection. For enumeration of bacillary load, specific portions of lungs and spleen were aseptically removed, weighed and homogenized separately in saline. Appropriate dilutions were plated in duplicates on MB7H11 agar plates and colonies were enumerated following incubation at 37°C for 3–4 weeks. The bacillary load was expressed as log_10_ CFU/organ. Visual scoring of gross lesions for the lungs, spleen and liver was carried out based on tissue involvement, areas of inflammation, number and size of tubercles and the extent of necrosis. The scores are graded from 1–4 based on the modified Mitchison scoring system [52]. For histopathological evaluation, specific portion of lungs and liver were removed and fixed in 10% buffered formalin. 5 μm thick sections of paraffin embedded tissues were stained with haematoxylin-eosin (H&E). The sections were examined for the granulomas and graded by a certified pathologist (who was blinded to the experimental groups) according to the criteria described previously by Lasco *et al* [53]. Briefly, granuloma with necrosis was given a score of 5, granuloma without necrosis was given a score of 2.5 and granuloma with fibrous tissue was given a score of 1. All granulomas in each section were scored and the scores were added up to obtain a total granuloma score of lungs or liver of each animal. In addition, sections were semi-quantitatively assessed for percentage of the section occupied by granuloma and this was expressed as granuloma fraction.

To evaluate *in vivo* growth kinetics and attenuation of MtbΔ*bioA* following intradermal administration, animals in the groups of five were intradermally injected with 1 x 10^6^ CFU of either *M*. *tuberculosis*, BCG or MtbΔ*bioA* in 100 μl of saline. Animals were euthanized by CO_2_ asphyxiation at 1, 3 and 6 weeks post inoculation. Bacillary enumeration, gross pathological evaluation and histopathological evaluation were carried out as described in the previous section. The bacillary load was expressed as log_10_ CFU/organ.

### Evaluation of protective efficacy of MtbΔ*bioA* against *M*. *tuberculosis* infection

Groups of guinea pigs (n = 6) were intradermally immunized with either (i) 1 x 10^6^ CFU of BCG (Danish strain) in 100 μl of saline or (ii) 1 x 10^6^ CFU of MtbΔ*bioA* in 100 μl of saline or (iii) two doses of 1 x 10^6^ CFU of MtbΔ*bioA* administered at 6-week interval (Δ*bioA*/Δ*bioA*). Control group (sham-immunized) guinea pigs received 100 μl of saline intradermally. Guinea pigs were challenged with aerosolized virulent *M*. *tuberculosis* Erdman strain (~50 bacilli in the lungs per animal) at 12 weeks post primary immunization and were euthanized by CO_2_ asphyxiation at 4 weeks post challenge. Organs were subjected to bacterial enumeration, gross pathological and histopathological evaluation as described in the previous section. Samples from the BCG-immunized animals were plated on MB-7H11 agar containing 2 μg/ml of thiophene-2-carboxylic acid hydrazide (Sigma) to inhibit the growth of residual BCG, if any.

### Statistical analysis

For comparison between the groups for the determination of bacillary load in the lungs or spleen and organ weight of experimental guinea pigs, unpaired *t*-test (two-tailed) or one-way analysis of variance (ANOVA) with the Tukey's multiple comparison test was employed, wherever appropriate. For comparison between the groups for the analyses of total granuloma score, Mann-Whitney test (two-tailed) was employed. Nonparametric Kruskal-Wallis test followed by the Dunn's multiple comparison test was employed for comparison between the groups of animals for the analysis of the gross pathological damage. Differences were considered significant when *p* <0.05. Prism software (GraphPad Software Inc., CA) was employed for generation of graphs and statistical analysis.

## Results

### Disruption of the *bioA* gene in *M*. *tuberculosis* and characterization of the mutant

**Fig 1:**
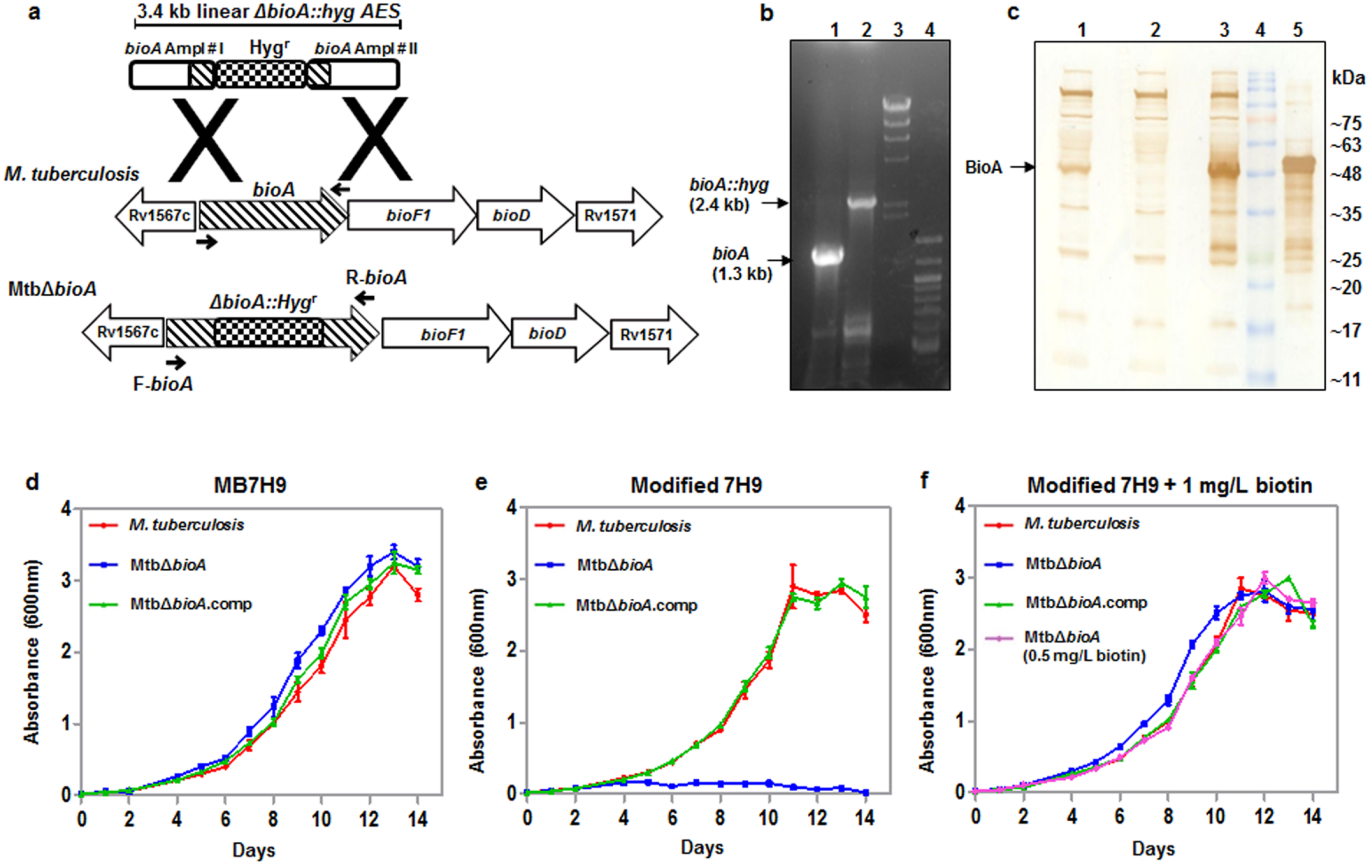
Confirmation of disruption of the *bioA* gene in *M*. *tuberculosis*. (a) Diagrammatic representation of the *bioA* locus in *M*. *tuberculosis* and MtbΔ*bioA*. The *bioA* gene is the first gene of an operon consisting of three downstream genes *bioF1*, *bioD* and Rv1571. The figure depicts the disruption of the *bioA* gene in MtbΔ*bioA* by homologous recombination. Arrows show the location of the *bioA* gene specific primers F-*bioA* and R-*bioA* employed for the confirmation of disruption of the *bioA* gene by PCR. (b) PCR based confirmation of disruption of *bioA* gene in MtbΔ*bioA*. A 1.3 kb PCR amplification product was obtained with the *M*. *tuberculosis* genomic DNA as template (lane 1) and 2.4 kb PCR amplification product was obtained with MtbΔ*bioA* genomic DNA as template (lane 2). λ*Hind*III DNA molecular mass marker and 100 bp ladder were loaded in lanes 3 and 4, respectively. (c) Confirmation of disruption of *bioA* in MtbΔ*bioA* by immunoblot analysis. 10 μg of cell lysate of *M*. *tuberculosis* (lane 1), MtbΔ*bioA* (lane 2) and MtbΔ*bioA*.comp (lane 3) strains were separated on a 12.5% polyacrylamide gel and immunoblot analysis was carried out with anti-BioA polyclonal antiserum. BioA (~48 kDa band) was detected in the cell lysate of the *M*. *tuberculosis* and MtbΔ*bioA*.comp. The disruption of *bioA* in MtbΔ*bioA* was confirmed by the absence of the ~48 kDa band in lane 2. Protein molecular weight marker and 150 ng of purified BioA were loaded in lanes 4 and 5, respectively. (d, e, f) Influence of disruption of the *bioA* gene on the growth of *M*. *tuberculosis* in culture media. *M*. *tuberculosis*, MtbΔ*bioA* and MtbΔ*bioA*.comp were cultured separately in (d) MB7H9 medium, (e) modified 7H9 medium (prepared without biotin) and (f) modified 7H9 medium supplemented with 1 mg/L of biotin. Growth at 37°C/200 rpm was monitored for 14 days by measuring the absorbance at 600 nm. MtbΔ*bioA* failed to grow in modified 7H9 medium and the growth was restored to wild type levels when modified 7H9 was supplemented with 1 mg/L or 0.5 mg/L of biotin. Two independent growth analysis experiments were carried out in duplicates and the values of absorbance are represented as the mean (±SE).

To generate the MtbΔ*bioA* mutant, a 3.4 kb linear *ΔbioA*::*hyg* AES was employed as described in the methods section. Disruption of the *bioA* gene in *M*. *tuberculosis* was confirmed by polymerase chain reaction (PCR) analysis by using *bioA* gene specific primers (Fig 1a and 1b, Table 1). A 1.3 kb PCR product was observed in the case of *M*. *tuberculosis* (Fig 1b). However, a 2.4 kb PCR product was observed in the case of MtbΔ*bioA* which corresponds with the replacement of 916 bp internal segment of the *bioA* gene with the ~ 2.0 kb hygromycin resistance cassette (Fig 1b). Further, *M*. *tuberculosis*, MtbΔ*bioA* and complement strain (MtbΔ*bioA*.comp) were subjected to immunoblot analysis with anti-BioA rabbit polyclonal antiserum (Fig 1c). In the *M*. *tuberculosis* cell lysate, BioA was detected as a ~48 kDa band which was absent from the cell lysate of MtbΔ*bioA* mutant (Fig 1c). Restoration of the ~48 kDa band was observed in the cell lysate of MtbΔ*bioA*.comp strain confirming the complementation of MtbΔ*bioA*. The results of PCR and immunoblot analysis confirm the disruption of the *bioA* gene in MtbΔ*bioA*.

Next, we compared the growth of *M*. *tuberculosis*, MtbΔ*bioA* and MtbΔ*bioA*.comp in MB7H9 media (which contains 0.5 mg/L of biotin) as well as in 7H9 media prepared without biotin (modified 7H9). We did not observe any difference in the growth of *M*. *tuberculosis*, MtbΔ*bioA* and MtbΔ*bioA*.comp in MB7H9 medium (Fig 1d). However, MtbΔ*bioA* failed to grow in modified 7H9 media (Fig 1e). The growth of MtbΔ*bioA* was completely restored when modified 7H9 media was supplemented with 1 mg/L or 0.5 mg/L of biotin (Fig 1f). MtbΔ*bioA*.comp strain demonstrated similar growth kinetics as *M*. *tuberculosis* in all the three culture conditions. These results demonstrate that the disruption of the *bioA* gene in *M*. *tuberculosis* renders the pathogen auxotrophic for biotin that can be complemented with the wild-type copy of the *bioA* gene.

### Biotin auxotroph of *M*. *tuberculosis* is highly attenuated in guinea pigs

**Fig 2:**
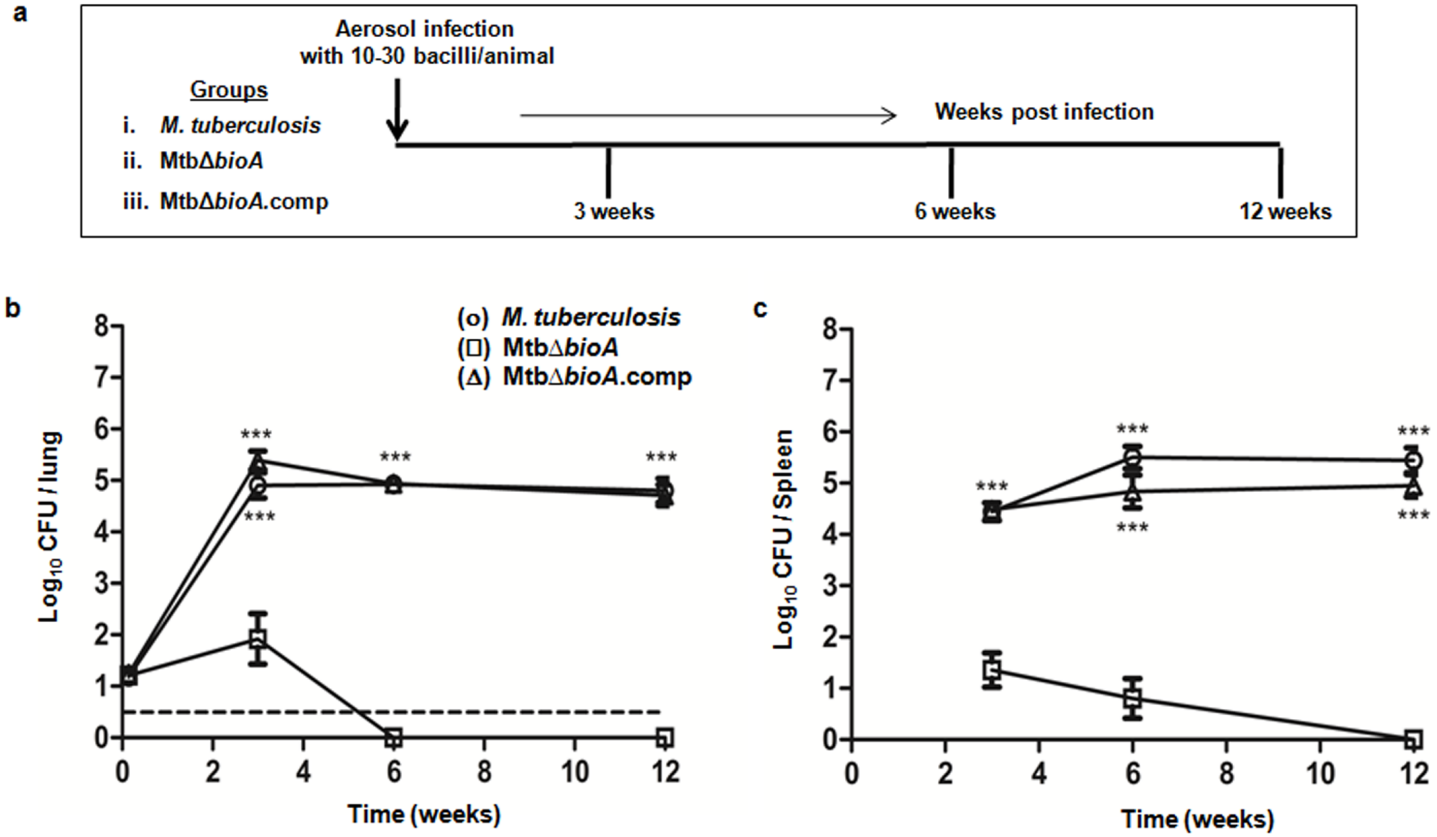
Influence of disruption of the *bioA* gene on the attenuation of *M*. *tuberculosis* in guinea pigs. (a) Experimental protocol for evaluating the influence of disruption of the *bioA* gene on the pathogenicity of *M*. *tuberculosis* in guinea pigs infected via aerosol. The figure shows the bacillary load in the (b) lungs and (c) spleen of guinea pigs at 3, 6 and 12 weeks post infection with *M*. *tuberculosis* (○), MtbΔ*bioA* (□) or MtbΔ*bioA*.comp (△) strains. Three animals per group were euthanized to determine the retained dose in the lungs following aerosol infection. Each data point at 3, 6, and 12 weeks represents the mean (±SE) of the log_10_ CFU/organ obtained with six guinea pigs per experimental group. Dotted line depicts the limit of detection for pulmonary bacillary load, meaning thereby that at 6 and 12 weeks post infection no bacilli were obtained from the total homogenate of half lung of MtbΔ*bioA* infected animals. No bacilli were obtained from the complete spleen homogenates of MtbΔ*bioA* infected animals at 12 weeks post infection. *** *p* <0.001 [unpaired *t*-test (two-tailed)] when compared with MtbΔ*bioA* infected animals.

In order to evaluate the effect of disruption of the *bioA* gene on the virulence of *M*. *tuberculosis*, guinea pigs were infected with *M*. *tuberculosis*, MtbΔ*bioA* or MtbΔ*bioA*.comp strains via the aerosol route (Fig 2a). *M*. *tuberculosis* infected animals demonstrated an increase in the bacterial burden during the initial phase of infection (Fig 2b and 2c). At 3 weeks post infection, the *M*. *tuberculosis* infected animals exhibited a high bacillary load of 4.90 log_10_ CFU in the lungs and 4.44 log_10_ CFU in the spleen (Fig 2b and 2c). Thereafter, the bacterial load remained steady in the lungs of *M*. *tuberculosis* infected animals. In the spleen, the bacillary load of *M*. *tuberculosis* stabilized after 6 weeks. Relative to *M*. *tuberculosis* infection, MtbΔ*bioA* infected animals showed significantly reduced ability to multiply in guinea pig organs (Fig 2b and 2c). At 3 weeks post infection, MtbΔ*bioA* infected animals exhibited around 10^3^ fold less bacillary load in the lungs and spleen, when compared with the bacillary load in *M*. *tuberculosis* infected animals (Fig 2b and 2c). Thereafter, the bacterial numbers in the lungs rapidly declined and no bacilli were recovered from the lungs of the animals infected with MtbΔ*bioA* at 6 weeks post infection (Fig 2b). In the spleen, MtbΔ*bioA* persisted in low numbers at 6 weeks and was gradually eliminated by 12 weeks post infection (Fig 2c). Thus, MtbΔ*bioA* exhibits an attenuated phenotype in guinea pigs. The bacterial burden observed in the lungs and spleen of animals infected with the MtbΔ*bioA*.comp strain was comparable to that of *M*. *tuberculosis* infected animals at all the timepoints screened. This demonstrated that the attenuation observed for the MtbΔ*bioA* strain was attributable to the disruption of the *bioA* gene.

### *bioA* mutant of *M*. *tuberculosis* is unable to cause tuberculous pathology in guinea pigs

At 3 weeks post aerosol infection, *M*. *tuberculosis* or MtbΔ*bioA*.comp infected animals exhibited moderate gross pathological damage of lungs and spleen with occasionally visible large tubercles (S2a Fig). Lungs of few of the animals infected with MtbΔ*bioA* showed scanty small sized lesions. Spleen and liver of animals infected with MtbΔ*bioA* showed minimal involvement with negligible gross pathological damage. At 6 and 12 weeks post infection, both *M*. *tuberculosis* and MtbΔ*bioA*.comp infected animals exhibited progressively worsening pulmonary as well as extra-pulmonary gross pathological damage (S2b and S2c Fig). However, at these timepoints, animals infected with MtbΔ*bioA* showed minimal involvement of lungs, spleen and liver with significantly less score when compared with the organs of *M*. *tuberculosis* or MtbΔ*bioA*.comp infected animals (S2b and S2c Fig). The comparison of mean weights of lungs and spleen of infected guinea pigs, as a measure of disease severity, demonstrated that MtbΔ*bioA* infected animals had significantly less mean spleen weight when compared with *M*. *tuberculosis* and MtbΔ*bioA*.comp infected animals at all the timepoints screened (S2d Fig). In addition, relative to *M*. *tuberculosis* and MtbΔ*bioA*.comp infected animals, a significant reduction in the mean lung weight of guinea pigs infected with MtbΔ*bioA* was observed by 12 weeks post infection (S2d Fig).

**Fig 3:**
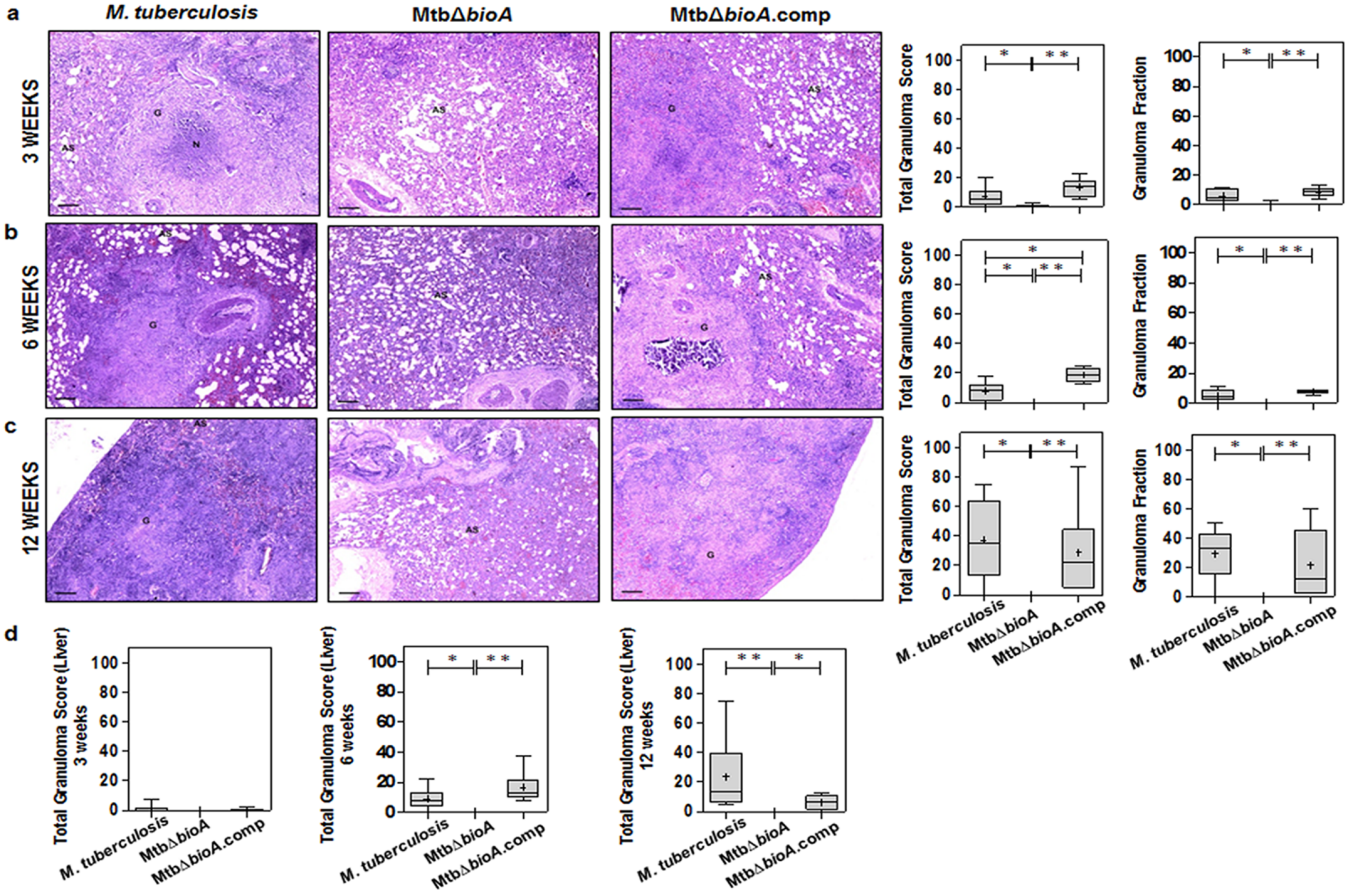
Histopathology of guinea pig tissues post aerosol infection with *M*. *tuberculosis*, MtbΔ*bioA* or MtbΔ*bioA*.comp strains. The figure depicts representative 40x magnification photomicrographs of formalin fixed, paraffin embedded and haematoxylin-eosin (H&E) stained 5 μm sections of lung tissue of guinea pigs aerogenically infected with *M*. *tuberculosis*, MtbΔ*bioA* or MtbΔ*bioA*.comp and euthanized at (a) 3 weeks, (b) 6 weeks and (c) 12 weeks post infection. AS, G and N denote alveolar spaces, granuloma and necrosis, respectively. The graphical representation of the total granuloma score and granuloma fraction are shown alongside by box plot of all six animals per group (the mean is represented by ‘+’, median value is denoted by horizontal line, box represents the inter quartile range and the minimum and maximum value is denoted by whiskers). (d) Total granuloma score for liver sections of infected animals (n = 6 animals per group) at 3, 6 and 12 weeks post infection. MtbΔ*bioA* infected animals showed negligible pulmonary as well as hepatic granulomatous pathological damage. *M*. *tuberculosis* and MtbΔ*bioA*.comp infected animals displayed increasing number of granulomas in the lungs as well as liver. The scale bars depict 200 μm. *p <0.05 and **p <0.01 (Mann-Whitney test, two-tailed).

Histopathological evaluation of lung tissues demonstrated that *M*. *tuberculosis* and MtbΔ*bioA*.comp infected animals showed progressively worsening granulomatous pathology in the lungs with granulomas composed of epithelioid cells and lymphocytes with occasional central necrosis (Fig 3a). MtbΔ*bioA* infected animals demonstrated significantly less total granuloma score of the lungs when compared to *M*. *tuberculosis* and MtbΔ*bioA*.comp infected animals at all the timepoints screened (Fig 3). At 3 weeks post infection, five out of six animals infected with MtbΔ*bioA* demonstrated minimal histopathological changes in lung parenchyma with scattered lymphocytic infiltration but without granulomatous lesions compared to infection with *M*. *tuberculosis* and MtbΔ*bioA*.comp, where granulomatous pathology was observed along with few necrotic granulomas (Fig 3a). No histopathological evidence of granuloma formation were observed at 6 and 12 weeks post infection with MtbΔ*bioA* (Fig 3b and 3c). Upon histopathological evaluation of the liver at 3, 6 and 12 weeks post infection, MtbΔ*bioA* infected animals did not show any evidence of granulomatous pathology in the liver while *M*. *tuberculosis* and MtbΔ*bioA*.comp infected animals showed progressive pathological damage of the hepatic tissue (Fig 3d). Taken together, the gross and histopathological evaluation of organs from MtbΔ*bioA* infected animals indicates that the *bioA* mutant of *M*. *tuberculosis* is unable to cause pathological damage in guinea pig organs.

### Difference in persistence of MtbΔ*bioA* and BCG following intradermal administration

**Fig 4:**
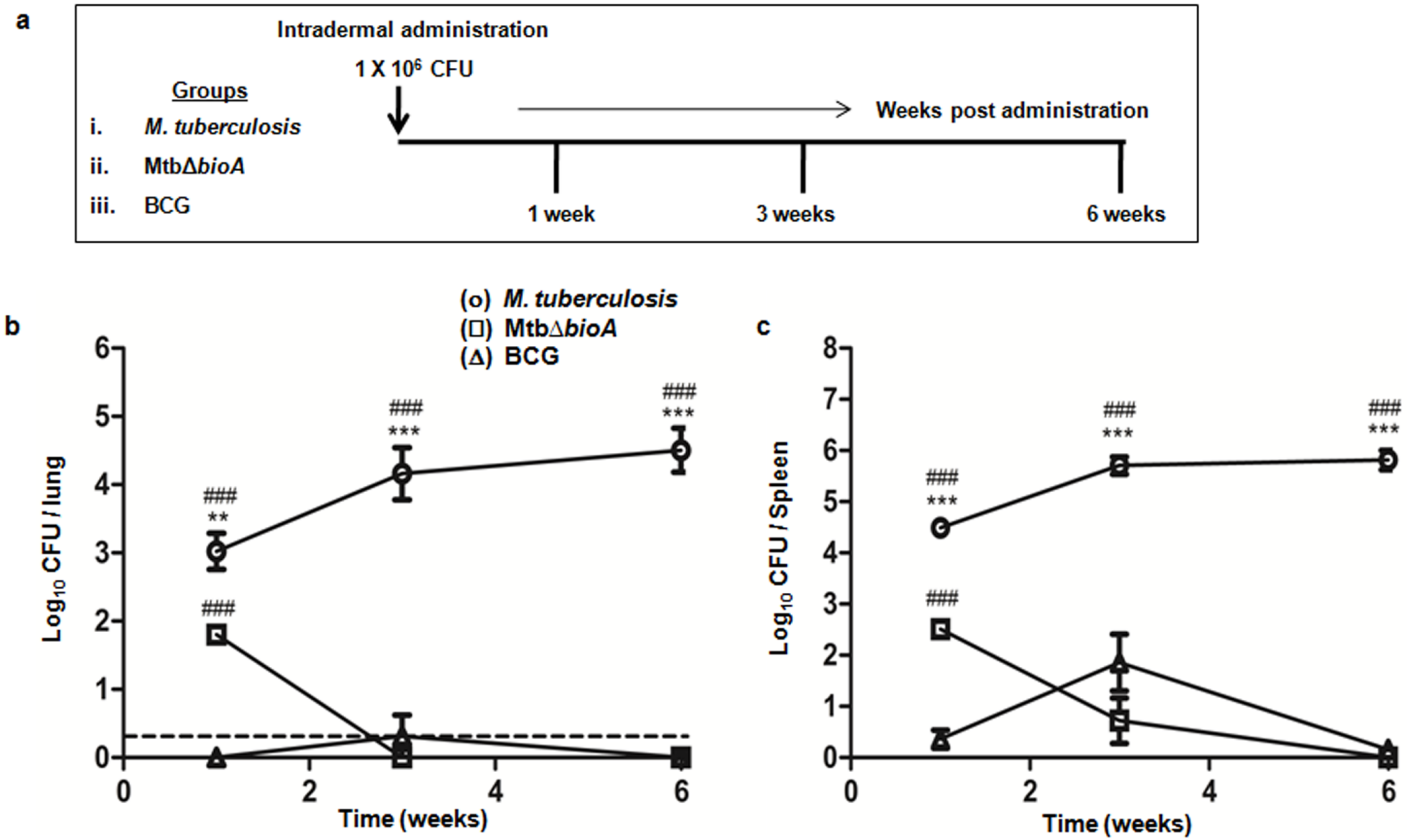
*In vivo* growth kinetics of MtbΔ*bioA* in guinea pigs following intradermal administration. (a) Experimental protocol for the evaluation of *in vivo* growth kinetics and attenuation of MtbΔ*bioA* in guinea pigs upon intradermal administration. The figure shows the bacillary load in the (b) lungs and (c) spleen of guinea pigs at 1, 3 and 6 weeks post inoculation with *M*. *tuberculosis* (○), MtbΔ*bioA* (□) or BCG (△). Each data point represents the mean (±SE) of the log_10_ CFU/organ obtained with five guinea pigs per experimental group per timepoint. Dotted line depicts the limit of detection for pulmonary bacillary load, meaning thereby that no bacterial colony was obtained from the total homogenate of half lung of all the MtbΔ*bioA* inoculated animals as well as in some of the BCG inoculated animals at 3 and 6 weeks post administration. No bacterial colony was obtained from the total spleen homogenates of MtbΔ*bioA* inoculated animals at 6 weeks post administration. **p <0.01 and ***p <0.001 [unpaired *t*-test (two-tailed)] when compared with animals injected with MtbΔ*bioA*. ^###^*p* <0.001 [unpaired *t*-test (two-tailed)] when compared with animals injected with BCG.

**Fig 5:**
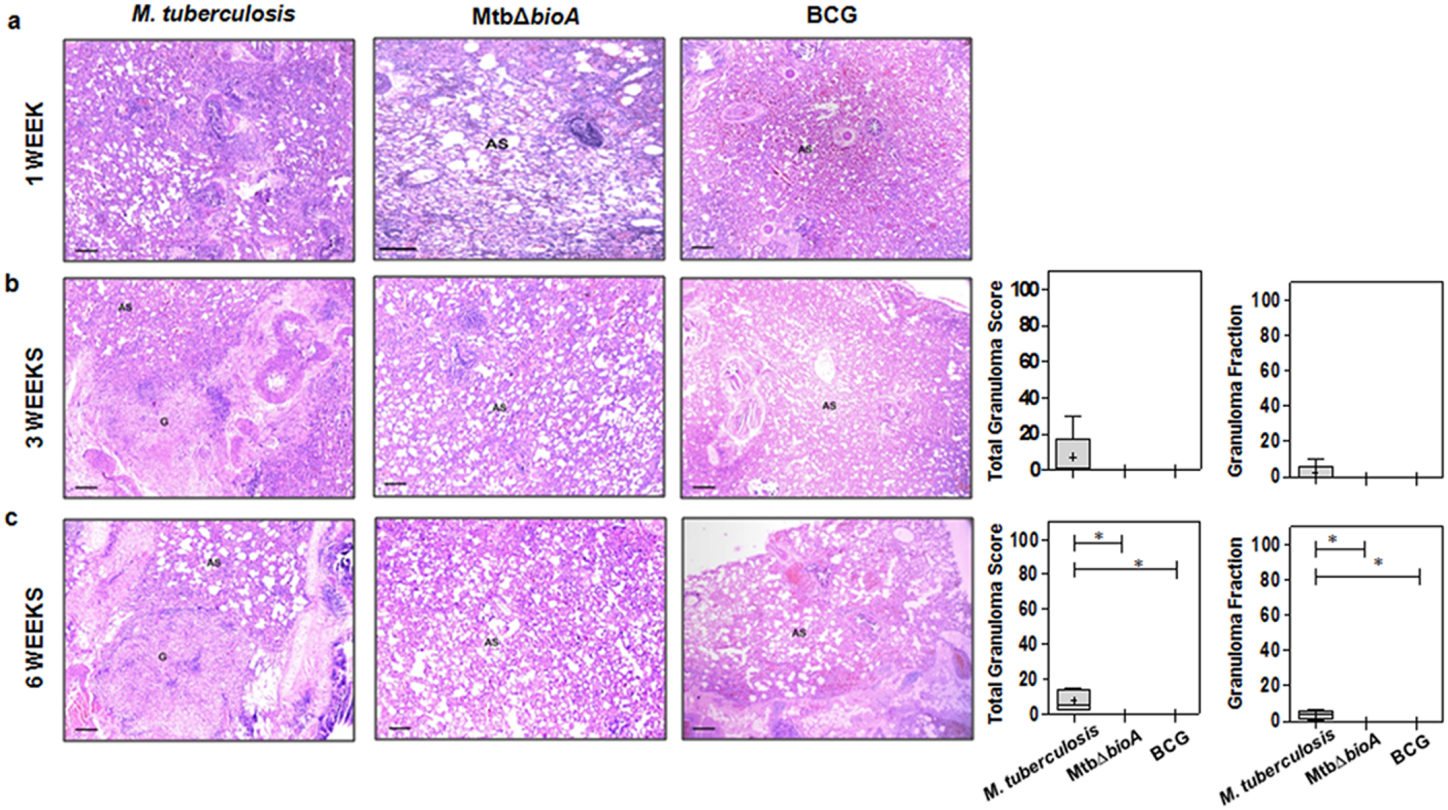
Histopathological analysis of guinea pig lungs post intradermal administration of *M*. *tuberculosis*, MtbΔ*bioA* or BCG. The figure depicts representative 40x magnification photomicrographs of formalin fixed, paraffin embedded and haematoxylin-eosin (H&E) stained 5 μm sections of lung tissue of guinea pigs intradermally inoculated with *M*. *tuberculosis*, MtbΔ*bioA* or BCG and euthanized at (a) 1 week, (b) 3 weeks and (c) 6 weeks post inoculation. AS denotes alveolar spaces and G denotes granuloma. The graphical representation of the total granuloma score and granuloma fraction are shown alongside by box plot of 5 animals per group (the mean is represented by ‘+’, median value is denoted by horizontal line, box represents the inter quartile range and the minimum and maximum value is denoted by whiskers). While animals inoculated with *M*. *tuberculosis* showed increasing pathological damage, the animals infected with MtbΔ*bioA* or BCG continued to exhibit no granulomatous pathology. The scale bars depict 200 μm. *p <0.05 (Mann-Whitney test, two-tailed).

Prior to the evaluation of vaccine efficacy, *in vivo* growth kinetics and attenuation of MtbΔ*bioA* was analyzed following intradermal administration in guinea pigs and was compared with *M*. *tuberculosis* and BCG (Fig 4a). Animals injected with *M*. *tuberculosis* exhibited an increase in the bacillary load in the lungs and spleen from 1 week to 3 weeks post inoculation after which the bacillary load stabilized (Fig 4b and 4c). However, relative to *M*. *tuberculosis* inoculated animals, MtbΔ*bioA* inoculated animals exhibited significantly reduced bacillary load [by 1.220 log_10_ CFU in the lungs (*p* = 0.0024) and by 1.985 log_10_ CFU in the spleen (*p* <0.0001)] as early as 1 week post injection (Fig 4b and 4c). The bacterial burden of MtbΔ*bioA* declined rapidly in both lungs and spleen. No bacilli could be recovered from the lungs and spleen of all of the MtbΔ*bioA* inoculated animals at 3 and 6 weeks post inoculation, respectively (Fig 4b and 4c). Animals inoculated with BCG showed the lowest bacillary load at 1 week post inoculation with no bacilli in the lungs and 0.36 log_10_ CFU in the spleen (Fig 4b and 4c). Subsequently, the bacillary load of BCG showed slight increase from 1 to 3 weeks post inoculation. The increase in bacillary load was more pronounced in the spleen (Fig 4c). Thereafter, the bacterial load declined and only one out of five animals inoculated with BCG showed 0.77 log_10_ CFU in the spleen while no bacilli were recovered from the lungs by 6 weeks post inoculation (Fig 4b and 4c). Thus, even though significantly higher (*p* <0.0001) bacillary load of MtbΔ*bioA* was observed in the organs when compared with BCG at 1 week post inoculation, the bacillary numbers of MtbΔ*bioA* rapidly declined to levels below that of BCG and were undetectable by 6 weeks. However, BCG showed some persistence in the spleen (Fig 4c). Histopathologically, at 3 and 6 weeks post intradermal administration, *M*. *tuberculosis* infected animals exhibited pathological damage of the lungs with granulomas composed of epithelioid cells and lymphocytes without any necrosis (Fig 5b and 5c). However, no evidence of granulomatous lesion were seen in the lungs of the animals inoculated with MtbΔ*bioA* or BCG at any of the timepoints screened (Fig 5).

### Vaccination of guinea pigs with MtbΔ*bioA* limits both pulmonary as well as extra-pulmonary multiplication of virulent *M*. *tuberculosis*

**Fig 6:**
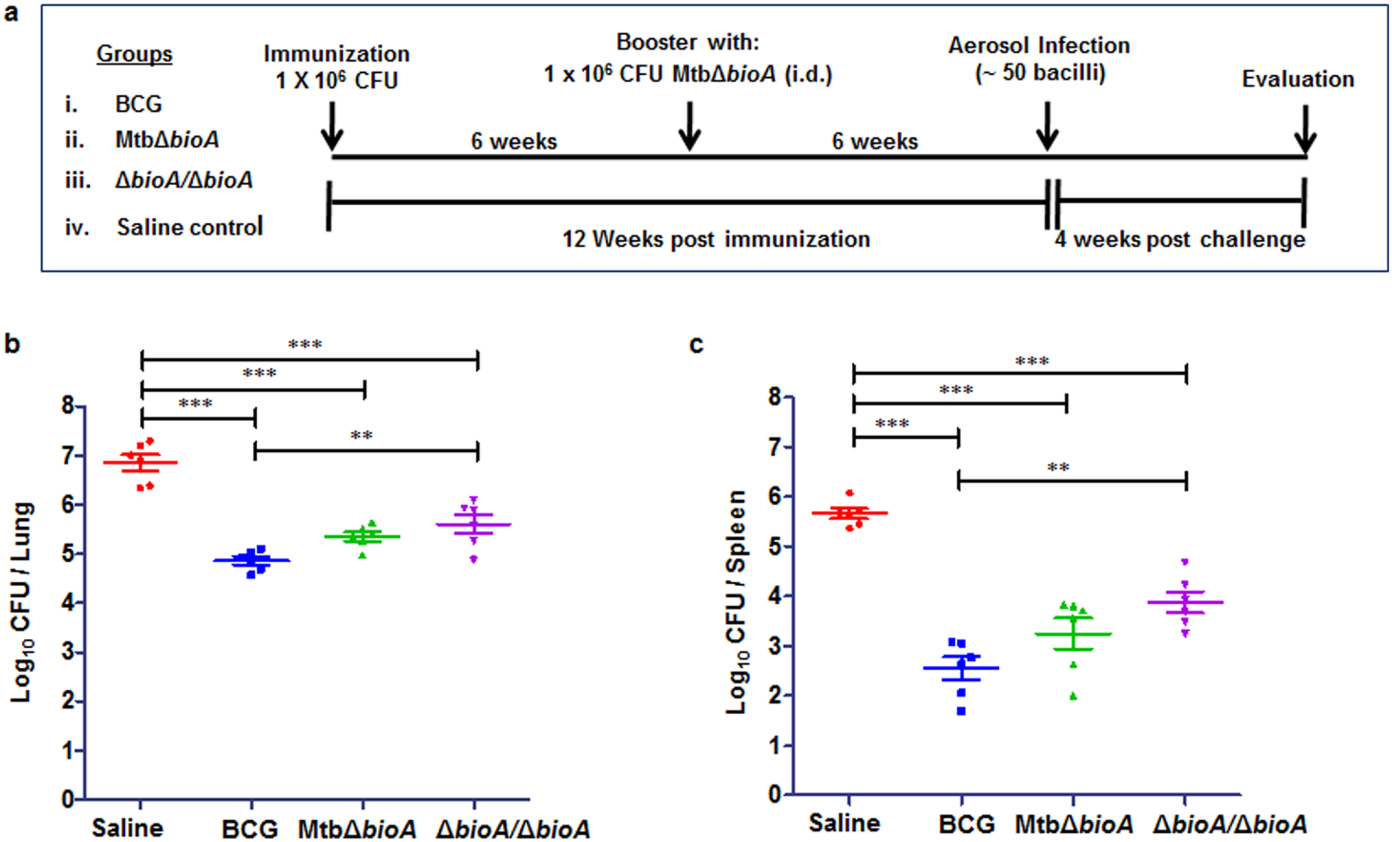
Evaluation of protective efficacy of MtbΔ*bioA* in guinea pig model of experimental tuberculosis. (a) Experimental protocol for assessing the protective efficacy of MtbΔ*bioA* against infection with virulent *M*. *tuberculosis* in guinea pigs. Guinea pigs (n = 6) were vaccinated with BCG or a single dose of MtbΔ*bioA* or two doses of MtbΔ*bioA* administered at 6-week interval (Δ*bioA/* Δ*bioA*). Vaccinated guinea pigs were challenged via the aerosol route with ~50 bacilli of virulent *M*. *tuberculosis* at 12 weeks from primary immunization and euthanized at 4 weeks post challenge. Sham-immunized animals were taken as control. (b, c) Enumeration of bacillary load in the lungs and spleen of vaccinated guinea pigs at 4 weeks post challenge. In both lungs and spleen, MtbΔ*bioA* imparted significant protection over sham-immunized controls. Booster dose with MtbΔ*bioA* failed to confer any improvement in protective efficacy imparted by single dose of MtbΔ*bioA*. Each data point represents the log_10_ CFU/ organ for an individual animal and the bar depicts mean (±SE) for each group. **p <0.01 and ***p <0.001 (one-way ANOVA followed by Tukey’s multiple comparison test).

Having assessed the attenuation of the MtbΔ*bioA* strain in guinea pigs via the aerosol as well as the intradermal route, we next evaluated the ability of this strain to induce protection against virulent *M*. *tuberculosis* challenge in guinea pig model of experimental TB. As shown in Fig 6a, guinea pigs were vaccinated intradermally (i.d.) with (i) BCG or (ii) MtbΔ*bioA*. In addition, we also evaluated the effect of boosting MtbΔ*bioA* with a second dose administered at 6-week interval (Δ*bioA*/Δ*bioA*). Guinea pigs were challenged with aerosolized virulent *M*. *tuberculosis* Erdman strain at 12 weeks post primary immunization and protective efficacy was evaluated four weeks post challenge. The sham-immunized guinea pigs showed the maximum infection with a bacillary load of 6.85 log_10_ CFU in the lungs and 5.66 log_10_ CFU in the spleen (Fig 6b and 6c). All the vaccinated groups demonstrated significant reduction in the bacillary load in the lungs and spleen, relative to bacterial burden in sham-immunized controls (Fig 6b and 6c). Vaccination with BCG resulted in the highest reduction in the bacterial load in the lungs (by 1.99 log_10_ CFU, *p* <0.0001) and in the spleen (by 3.114 log_10_ CFU, *p* <0.0001), when compared with sham-immunized controls. MtbΔ*bioA* vaccinated animals exhibited efficient control over the multiplication of virulent *M*. *tuberculosis* in the lungs and significantly reduced the bacillary burden by 1.504 log_10_ CFU (*p* <0.0001) when compared with sham-immunized animals (Fig 6b). Additionally, vaccination with MtbΔ*bioA* effectively controlled the dissemination of the challenge strain to the spleen resulting in significant reduction in bacillary burden (by 2.41 log_10_ CFU, *p* <0.0001) in the spleen when compared with the sham-immunized animals (Fig 6c). Importantly, even though vaccination with MtbΔ*bioA* imparted protection that was less when compared with the protection imparted by BCG in both lungs and spleen, the difference was not statistically significant (Fig 6b and 6c). When animals were vaccinated with two doses of MtbΔ*bioA* (Δ*bioA/*Δ*bioA*), the protection imparted was lower though not statistically different relative to single immunization (Fig 6b and 6c). Moreover, the protection imparted by Δ*bioA/*Δ*bioA* vaccine regimen was significantly poor when compared with BCG vaccinated animals in lungs as well as spleen (Fig 6b and 6c). Thus, our observations indicate that single administration of MtbΔ*bioA* is able to efficiently control the multiplication of challenge strain at the primary site of infection as well as exhibit significant control on the hematogenous spread of virulent *M*. *tuberculosis*.

### Vaccination with MtbΔ*bioA* protects against pathological damage

**Fig 7:**
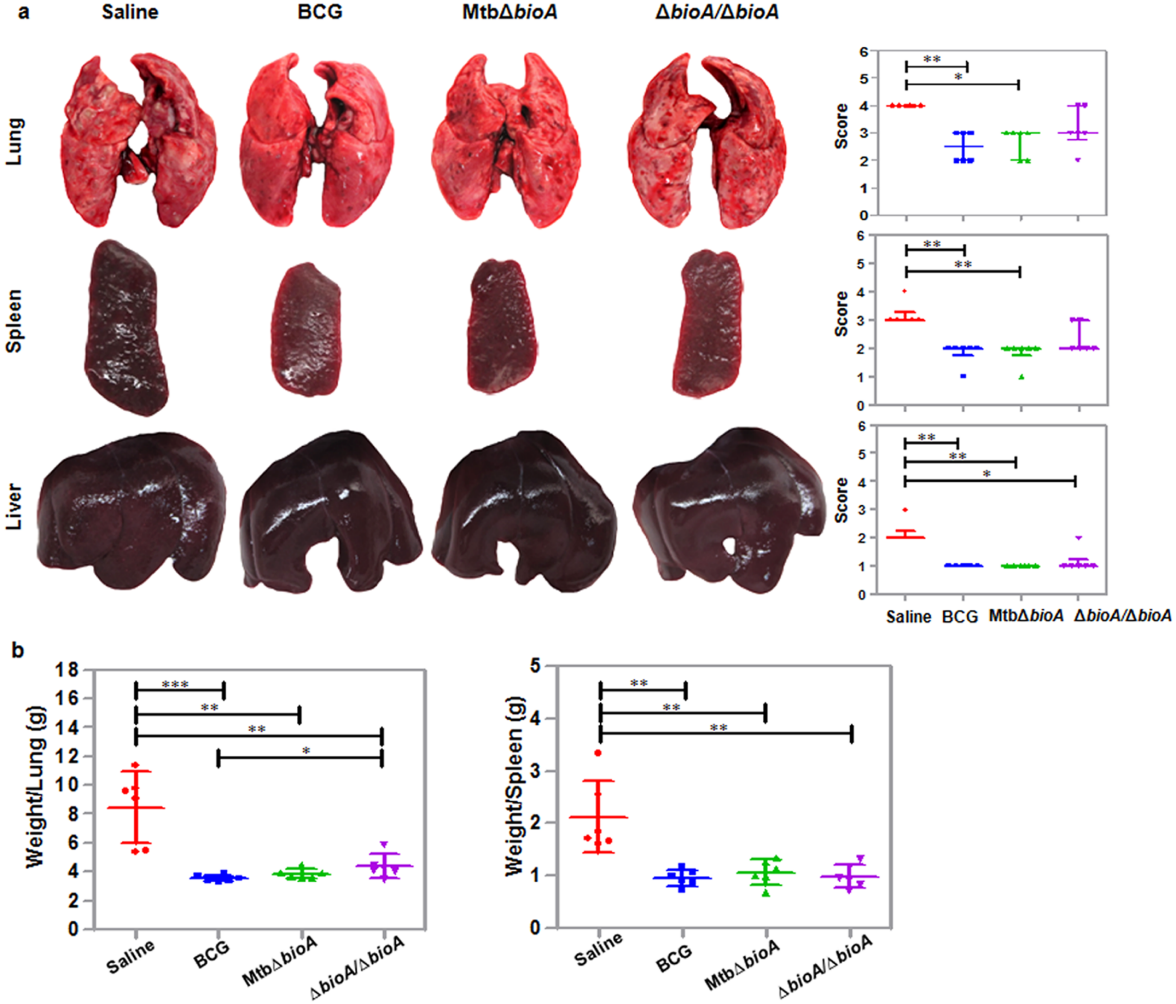
Evaluation of the gross pathological damage of the organs of vaccinated guinea pigs at 4 weeks post challenge. (a) The figure depicts representative photographs of lungs, spleen and liver of guinea pigs belonging to different groups of vaccination at 4 weeks post challenge. Graphical representation of gross pathological scores assigned to the lungs, spleen and liver of guinea pigs euthanized at 4 weeks post challenge is represented alongside. Each data point represents the score assigned to an individual animal. The bar depicts median (± interquartile range) for each group. * *p* <0.05 and **p <0.01 (Kruskal-Wallis test followed by the Dunn's multiple comparison test). (b) The graphical representation of organ (lungs and spleen) weights of guinea pigs at 4 weeks post challenge. The bar depicts mean (± SD) for each group. * *p* <0.05, **p <0.01 and ***p <0.001 [unpaired (two-tailed) *t*-test].

In addition to the bacillary load, pathological damage is an important read out for pronouncing the efficacy of a candidate vaccine. Upon gross pathological evaluation, the maximum damage was noted in sham-immunized animals with numerous small as well as large necrotic tubercles spread throughout the lungs (Fig 7a). Extensive extra-pulmonary damage was seen in the spleen and liver of sham-immunized animals with numerous small tubercles present throughout the organs and marked splenomegaly. BCG vaccinated animals showed significant reduction in pathological damage of lungs, spleen and liver when compared with sham-immunized animals (Fig 7a). Lungs of BCG vaccinated animals demonstrated occasional large and countable number of small tubercles while spleen appeared normal in size with a moderate number of small tubercles (Fig 7a). Importantly, MtbΔ*bioA* vaccinated animals also showed significant reduction in gross pathological damage of lungs, spleen and liver when compared with the sham-immunized animals (Fig 7a). Lungs of MtbΔ*bioA* vaccinated animals showed a countable number of discrete small and large sized tubercles and scanty coalescent tubercles. Gross pathological scores of spleens of MtbΔ*bioA* vaccinated animals were similar to BCG vaccinated animals. Animals belonging to the Δ*bioA*/Δ*bioA* group showed maximum pathology among all the vaccinated groups of animals with numerous small and large tubercles in the lungs and moderate involvement of spleen with occasional large tubercles. Animals belonging to all the vaccinated groups showed minimal hepatic damage. The increase in weight of lungs and spleen is frequently associated with severity of disease in infected guinea pigs [54]. The mean organ (lungs and spleen) weights of guinea pigs belonging to all the three vaccinated groups were significantly lower than the mean organ weight of sham-immunized guinea pigs (Fig 7b). BCG and MtbΔ*bioA* vaccinated animals demonstrated comparable mean lung weights. However, the mean lung weight of Δ*bioA*/Δ*bioA* vaccinated animals was significantly higher (*p* = 0.0367) as compared to BCG vaccinated animals (Fig 7b). The mean spleen weights of guinea pigs belonging to all the vaccinated groups were comparable (Fig 7b).

**Fig 8:**
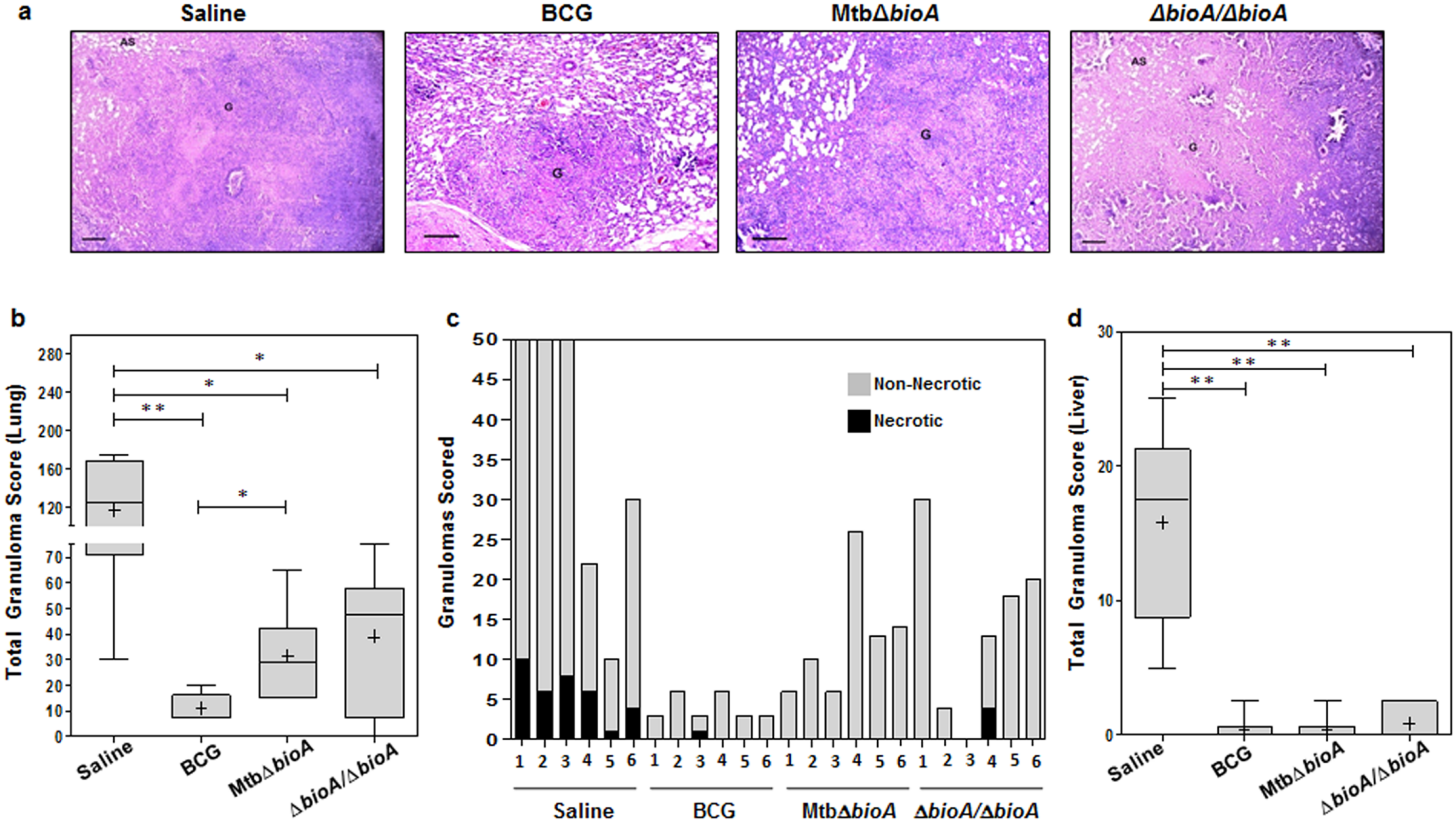
Histopathological analysis of vaccinated animals at 4 weeks post challenge. (a) The figure depicts representative 40x magnification photomicrographs of formalin fixed, paraffin embedded and haematoxylin-eosin (H&E) stained 5 μm sections of lung tissue of guinea pigs (n = 6) vaccinated with BCG, MtbΔ*bioA*, two doses of MtbΔ*bioA* (Δ*bioA*/Δ*bioA*) or saline and euthanized at 4 weeks post challenge. The scale bars depict 200 μm. AS and G denote alveolar spaces and granuloma, respectively. (b) The graphical representation of the total granuloma score of the lung sections is shown by box plot of 6 animals per group (the mean is represented by ‘+’, median value is denoted by horizontal line, box represents the inter quartile range and the minimum and maximum value is denoted by whiskers). *p <0.05 and **p <0.01 (Mann-Whitney test, two tailed). (c) The figure depicts the graphical representation of the number of granulomas that were scored in lung section from each animal in a group. The black region depicts the number of necrotic granulomas out of the total number of granulomas seen in lung section from each animal. (d) The graphical representation of the total granuloma score of the liver sections of 6 animals per group is shown by box plot (the mean is represented by ‘+’, median value is denoted by horizontal line, box represents the inter quartile range and the minimum and maximum value is denoted by whiskers). **p <0.01 (Mann-Whitney test, two tailed).

Histopathological evaluation and granuloma scoring of the lungs and liver tissue of the experimental animals demonstrated that the organs from sham-immunized animals had the maximum total granuloma score with a large number of necrotic granulomas (Fig 8). Significant reduction in the total granuloma score was observed in the lungs of the animals belonging to BCG, MtbΔ*bioA* and Δ*bioA*/Δ*bioA* vaccinated group when compared with the sham-immunized animals, indicating reduction in pulmonary granulomatous pathology (Fig 8b). The least total granuloma score of lungs was observed for the BCG vaccinated animals (Fig 8b). MtbΔ*bioA* vaccinated animals exhibited significantly higher (*p* = 0.0173) average total granuloma score when compared with the animals vaccinated with BCG (Fig 8b). Importantly, however, no necrotic granulomas were seen in the lung sections of any of the animals vaccinated with MtbΔ*bioA*, which was in contrast to that observed for BCG vaccinated animals and animals vaccinated with two doses of MtbΔ*bioA* (Fig 8c). Evaluation of histopathological damage of the liver sections demonstrated that the maximum pathological damage of liver occurred in the sham-immunized animals (Fig 8d). BCG as well as MtbΔ*bioA* vaccinated animals showed comparable hepatic total granuloma score which was significantly reduced (*p* = 0.005) when compared with sham-immunized animals (Fig 8d). Δ*bioA*/Δ*bioA* vaccinated animals showed marginally higher total granuloma score of the liver sections when compared with BCG or MtbΔ*bioA* vaccinated animals.

Moreover, in order to confirm that the vaccine strain does not get reactivated upon virulent *M*. *tuberculosis* infection and contribute towards the bacillary load observed at 4 weeks post challenge, all the lung and spleen homogenates from MtbΔ*bioA* and Δ*bioA*/Δ*bioA* vaccinated groups were plated on agar media containing hygromycin. No bacilli were seen in any of the plates following incubation at 37°C for 3–4 weeks, thus confirming that reactivation of vaccine strain does not occur.

## Discussion

Novel TB vaccines based on attenuated *M*. *tuberculosis* strains follow the Pasteurian approach where the vaccine strain is a modified form of the pathogen minus its disease-causing ability. Attenuated *M*. *tuberculosis* strains have shown promise as potential BCG replacement vaccines. In this study, we show that biotin biosynthesis is essential for the growth of *M*. *tuberculosis in vitro* as well as in guinea pig tissues. Further, we provide evidence that the attenuated biotin auxotroph of *M*. *tuberculosis* has the potential to generate protection against virulent *M*. *tuberculosis* challenge.

We generated a *bioA* gene mutant of *M*. *tuberculosis* and demonstrated its inability to grow in biotin deficient modified 7H9 media. The complementation of MtbΔ*bioA* with the full-length *bioA* gene restored its ability to grow in the absence of external biotin supplementation. The MtbΔ*bioA* strain was rapidly eliminated from the lungs and spleens of guinea pigs irrespective of the route of administration. Following aerosol infection with MtbΔ*bioA*, no bacilli were recovered from the lungs at 6 weeks post infection and from the spleen at 12 weeks post infection. Limited survival of the biotin auxotroph in the host tissue was concomitantly associated with negligible granulomatous pathology. Additionally, following the intradermal administration of MtbΔ*bioA* in guinea pigs, the bacillary numbers rapidly declined and were undetectable in the lungs by 3 weeks and in spleen by 6 weeks post inoculation. The *in vivo* growth kinetics of MtbΔ*bioA* following intradermal administration was different from that observed for the standard TB vaccine strain, BCG. Importantly, however, no evidence of granulomatous pathology was observed for MtbΔ*bioA* as well as BCG at any of the timepoints screened. These results indicate that the auxotrophic mutant MtbΔ*bioA* either doesn’t have access to biotin available from the host or it is insufficient to support the growth of the mutant, thereby rendering it attenuated for growth and virulence in guinea pigs. Our attenuation studies in guinea pigs corroborate with earlier observations of attenuated phenotypes of *M*. *smegmatis*, *M*. *marinum* and *M*. *tuberculosis bioA* gene mutants in mice or zebrafish [26–28, 30]. Though mice and guinea pig both have been extensively used for virulence as well as vaccine evaluation studies against tuberculosis, various aspects of the disease are represented to varied levels in the different animal models of experimental tuberculosis [44]. Thus, the *in vivo* kinetics and attenuation of a strain may vary depending on the animal model of evaluation such as an attenuated *M*. *tuberculosis* mutant in SigH (MtbΔ*sigH*) was shown to have contrasting replication capability in mice and primates [55, 56]. Our findings reconfirm the auxotrophic nature of MtbΔ*bioA* mutant and are in agreement with the conclusions drawn by Park *et al*. who reported the inability of a *bioA* gene mutant of *M*. *tuberculosis* to grow in Sauton’s liquid media and also demonstrated the dependence of *M*. *tuberculosis* on biotin biosynthesis for its growth and survival following aerosol infection of mice [30]. Further detailed study of the biodistribution of the MtbΔ*bioA* strain to organs such as lymph nodes, liver, kidney and brain (in addition to lungs and spleen) would help generate knowledge required for appropriate downstream development of this strain.

Importantly, since MtbΔ*bioA* persists in the host tissue during the early phase of infection, especially in the spleen, we evaluated its ability to generate protection against virulent *M*. *tuberculosis* challenge. Although preliminary, our short-term vaccine evaluation study in guinea pigs demonstrated that the attenuated biotin auxotroph of *M*. *tuberculosis* has the potential to impart protection. MtbΔ*bioA* vaccinated animals demonstrated significant reduction in the bacillary load in the lungs and spleen, when compared with sham-immunized animals. Vaccination with MtbΔ*bioA* could not reduce the bacterial burden to the same extent as in animals vaccinated with BCG. However, the difference was not statistically significant. Importantly, as opposed to the other vaccinated groups, none of the animals vaccinated with a single dose of MtbΔ*bioA* showed necrotic granulomas. Maximum necrosis was observed in sham-immunized animals. Necrotic granulomas have been associated with immunopathogenesis and necrosis can damage airway lining leading to bacterial transmission [57]. Thus, vaccination with a single dose of MtbΔ*bioA* demonstrated the ability to protect against pathological damage. However, administration of booster dose of MtbΔ*bioA* resulted in off-putting effects on the protection afforded by a single dose of MtbΔ*bioA*. Similar observations have been noted in other studies wherein the repeat administration of mycobacteria resulted in the abrogation of protection [14, 58–60]. The explanation for such an observation seems to be derived from Koch phenomenon, wherein repeat exposure to mycobacteria or its antigens result in a tissue damaging heightened inflammatory response [58, 61].

Several studies have compared the protective capacities of live attenuated *M*. *tuberculosis* mutants with BCG and have demonstrated varying degrees of success. Vaccine candidates such as MTBVAC, showed significantly superior protection when compared with BCG in short-term evaluation in mice and was found to be safe and immunogenic in healthy adults volunteers [9, 17, 62, 63]. Vaccination with *M*. *tuberculosis phoP* mutant strain, the prototype for MTBVAC, resulted in protection similar to BCG in mice and guinea pig in short term studies while demonstrating significantly better protection than BCG in guinea pig survival assay. [11]. Recently, mucosal vaccination with a *sigH* mutant of *M*. *tuberculosis* was shown to generate high levels of protection in rhesus macaques, when compared with BCG [64]. On the other hand, certain attenuated vaccine constructs such as a *M*. *tuberculosisΔpanCD*, *M*. *tuberculosisΔlysA*, *M*. *tuberculosisΔlysApanCD* and *M*. *tuberculosisΔleuDpanCD* were found to confer protection comparable to BCG [12, 14, 15, 65]. Interestingly, correlation of *in vivo* persistence of an attenuated *M*. *tuberculosis* strain with its ability to impart protection remains largely unknown. *M*. *tuberculosisΔpanCD* mutant persists for months *in vivo* and the persistence of the strain is thought to contribute towards the generation of protection comparable to BCG [15]. On the other hand, a mutant like *M*. *tuberculosisΔlysApanCD* shows rapid decline in bacillary numbers *in vivo* but generates protection comparable to BCG [65]. For such highly attenuated strains the initial interaction of the vaccine antigens with the immune system appears to be important for generating appropriate response and conferring protection [65]. Our data on the MtbΔ*bioA* strain highlight the vaccinogenic potential of highly attenuated *M*. *tuberculosis* strains against virulent *M*. *tuberculosis* challenge.

MtbΔ*bioA* provides no greater protection than BCG in the short-term vaccine efficacy study in guinea pigs employed in this work. Short-term protective efficacy studies are considered discriminative, predictive of survival and enable quick screening of active/inactive strains especially when compared with unvaccinated animals [66]. However, more stringent and appropriately powered survival study would be critical to determine the efficacy of the MtbΔ*bioA* strain as compared to BCG. Importantly, inspite of a short period of persistence in guinea pig tissue, MtbΔ*bioA* demonstrated the ability to protect against virulent *M*. *tuberculosis* Erdman challenge. These results encourage further studies on the MtbΔ*bioA* mutant. Potential strategies may include mucosal vaccination with MtbΔ*bioA*. Recently, mucosal vaccination with MtbΔ*sigH* was shown to induce strong central memory responses in the lungs resulting in the generation of significant protection from a lethal challenge in rhesus macaques [67]. Further, MtbΔ*bioA* strain can be modified by overexpressing immunodominant antigens that might boost the immune response. In addition, it is recommended by the regulatory authorities that there should be at least two independent mutations for live attenuated *M*. *tuberculosis* based vaccines in order to minimize the probability of reversion to wild type phenotype [68]. MtbΔ*bioA* strain can be further modified by introducing additional mutations to enhance its safety profile. Our efforts in this direction are in progress. Moreover, the protection imparted by MtbΔ*bioA* may be enhanced by combining the attenuating biotin auxotrophy with mutations that impact pathways involved in immunomodulation. Inactivating the *secA2* gene in *M*. *tuberculosis* has been shown to enhance T cell priming [10]. Combining highly attenuating lysine auxotrophy with *secA2* mutation (*M*. *tuberculosisΔsecA2ΔlysA*) has been shown to result in a safe and immunogenic vaccine that imparts significantly higher protection when compared with BCG [69]. In this study, the MtbΔ*bioA* strain has been shown to be protective against challenge with *M*. *tuberculosis* Erdman strain. The possibility of strain-specific response cannot be ruled out and it would be worthwhile to test its potential against challenge with clinical *M*. *tuberculosis* strains. Additionally, some of the promising attenuated *M*. *tuberculosis* mutants, such as MTBVAC and MtbΔ*sigH* are derived from clinical isolates of *M*. *tuberculosis* and have been shown to be safe, immunogenic and efficacious [9, 56]. Thus, it would be interesting to employ biotin auxotrophy to construct attenuated strains of clinical *M*. *tuberculosis* isolates and evaluate their vaccine efficacy against TB. Nevertheless, this work highlights the potential of biotin auxotrophy as a promising starting point for the development of novel attenuated strains as vaccines against TB.

## Supporting information

S1 Fig Curing of pJV53 from MtbΔ*bioA*. (a) Figure depicts the pictorial representation of the protocol of subsequent sub-culturing employed for the curing of plasmid pJV53 from MtbΔ*bioA*. MtbΔ*bioA* was sub-cultured in MB7H9 media in the absence of kanamycin and appropriate dilutions of tertiary culture were plated on MB7H11 agar without kanamycin and incubated at 37°C for 3–4 weeks. (b) Screening of colonies for the loss of pJV53. Individual colonies obtained on the agar plates were screened for the loss of pJV53 by patching on MB7H11 agar plates with or without kanamycin.
(TIF)

S2 Fig Evaluation of the gross pathology of the organs of guinea pig infected with *M*. *tuberculosis*, MtbΔ*bioA* or MtbΔ*bioA*.comp strains via aerosols. The figure depicts representative photographs of lungs, spleen and liver of guinea pigs following aerosol infection with *M*. *tuberculosis*, MtbΔ*bioA* or MtbΔ*bioA*.comp strains at (a) 3 weeks, (b) 6 weeks and (c)12 weeks post infection. Graphical representation of gross pathological scores assigned to the lungs, spleen and liver of each guinea pig at a particular time point is represented alongside. Each data point represents the score assigned to an individual animal. The bar depicts median(±interquartile range) for each group. *p <0.05 and **p <0.01 (Kruskal-Wallis test followed by Dunn's multiple comparison test). (d) The graphical representation of the organ (lungs and spleen) weights of guinea pigs at 3, 6 and 12 weeks post aerosol infection. Each data point represents the organ weight of an individual animal. The bar depicts mean (± SD) for each group. * *p* <0.05 and **p <0.01 [unpaired (two-tailed) *t*-test].
(TIF)
