# Supplementary figures and images for: bioA mutant of Mycobacterium tuberculosis shows severe growth defect and imparts protection against tuberculosis in guinea pigs
Source: PLoS One. 2017 Jun 28;12(6):e0179513. doi: 10.1371/journal.pone.0179513 (PMC5489182; doi:10.1371/journal.pone.0179513)

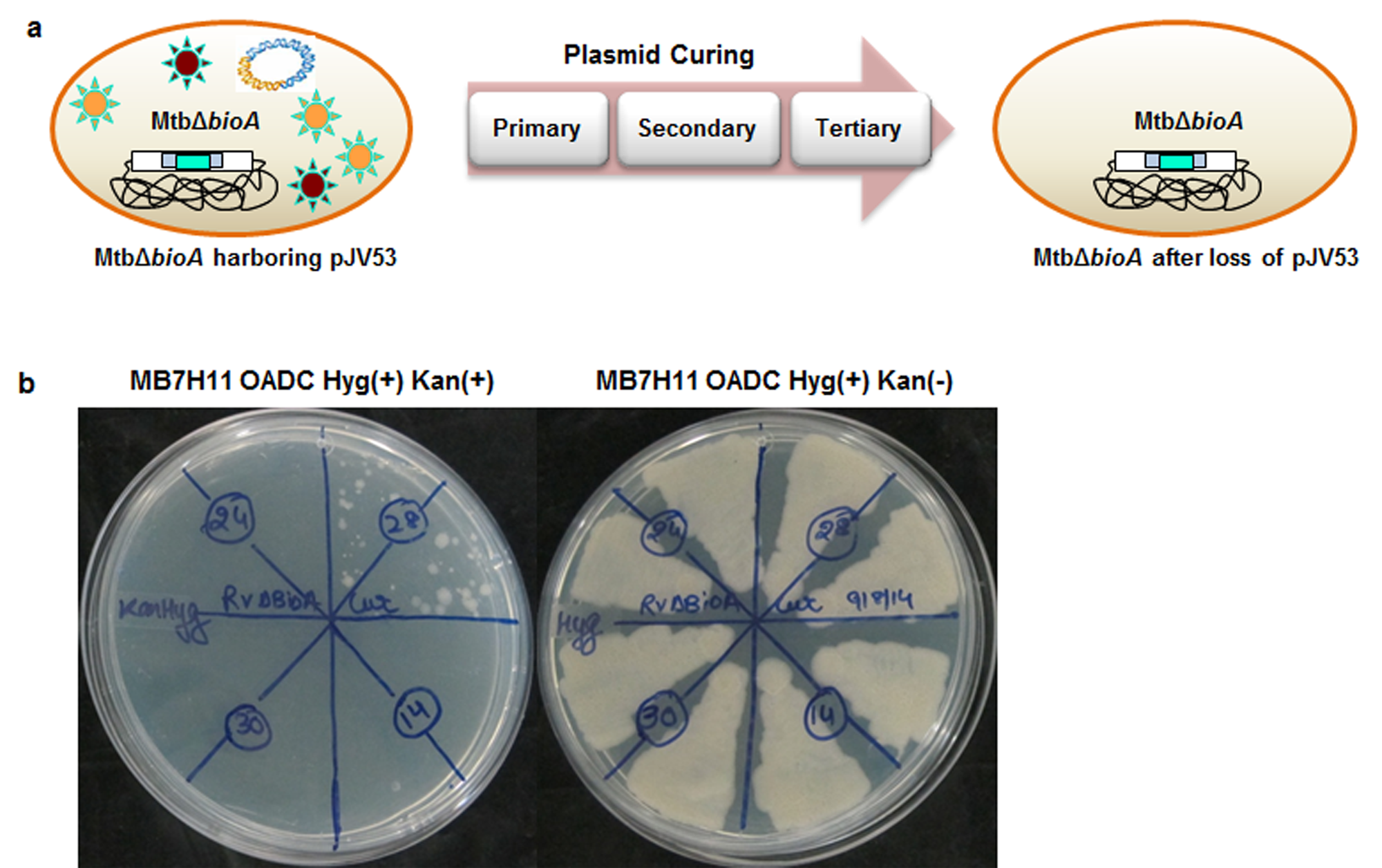

Supplement: S1 Fig — (a) Figure depicts the pictorial representation of the protocol of subsequent sub-culturing employed for the curing of plasmid pJV53 from MtbΔbioA. MtbΔbioA was sub-cultured in MB7H9 media in the absence of kanamycin and appropriate dilutions of tertiary culture were plated on MB7H11 agar without kanamycin and incubated at 37°C for 3–4 weeks. (b) Screening of colonies for the loss of pJV53. Individual colonies obtained on the agar plates were screened for the loss of pJV53 by patching on MB7H11 agar plates with or without kanamycin. (TIF) [file pone.0179513.s001.tif]

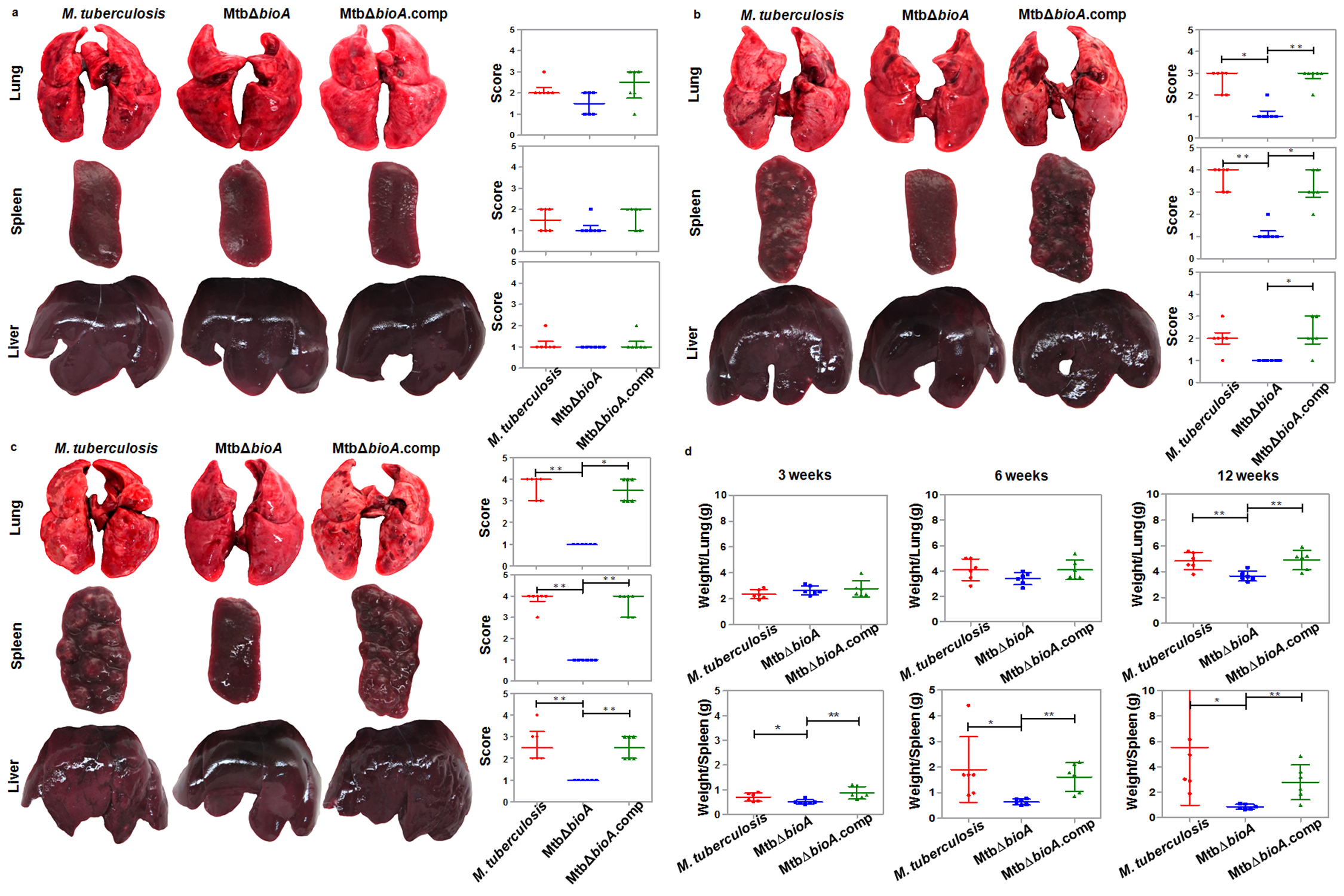

Supplement: S2 Fig — The figure depicts representative photographs of lungs, spleen and liver of guinea pigs following aerosol infection with M. tuberculosis, MtbΔbioA or MtbΔbioA.comp strains at (a) 3 weeks, (b) 6 weeks and (c)12 weeks post infection. Graphical representation of gross pathological scores assigned to the lungs, spleen and liver of each guinea pig at a particular time point is represented alongside. Each data point represents the score assigned to an individual animal. The bar depicts median(±interquartile range) for each group. *p <0.05 and **p <0.01 (Kruskal-Wallis test followed by Dunn's multiple comparison test). (d) The graphical representation of the organ (lungs and spleen) weights of guinea pigs at 3, 6 and 12 weeks post aerosol infection. Each data point represents the organ weight of an individual animal. The bar depicts mean (± SD) for each group. * p <0.05 and **p <0.01 [unpaired (two-tailed) t-test]. (TIF) [file pone.0179513.s002.tif]
